# The Determinants of Delays and Their Impact on Clinical End Points in Acute ST-Segment Elevation Myocardial Infarction: A Single-Center Experience

**DOI:** 10.3390/medicina61030447

**Published:** 2025-03-04

**Authors:** Kardelen Ohtaroglu Tokdil, Hasan Tokdil, Eser Durmaz, Bilgehan Karadag, Burcak Kilickiran Avci, Baris Ikitimur, Emre Ozmen, Alpin Mert Tekin, Betul Zehra Pirdal, Zeki Ongen

**Affiliations:** 1 Division of Cardiology, Cerrahpasa Medical Faculty, Istanbul University-Cerrahpasa, 34098 Istanbul, Turkey; durmazeser@hotmail.com (E.D.); karadag@istanbul.edu.tr (B.K.); burcakdr@yahoo.com (B.K.A.); ikitimur@gmail.com (B.I.); dremreozmen@yahoo.com (E.O.); dr.alpintekin@gmail.com (A.M.T.); z_ongen@yahoo.com.tr (Z.O.); 2 Division of Cardiology, Zonguldak Caycuma State Hospital, 67900 Zonguldak, Turkey; hasantokdil@gmail.com; 3 Division of Public Health, Cerrahpasa Medical Faculty, Istanbul University-Cerrahpasa, 34098 Istanbul, Turkey; bzehrapirdal@gmail.com

**Keywords:** ST-segment elevation myocardial infarction, delay time, primary percutaneous coronary intervention

## Abstract

*Background and Objectives*: The purpose of this study was to determine the factors that cause delay time in patients admitted to the hospital with STEMI. In addition, the effect of this delay on the patient’s prognosis has also been investigated. *Materials and Methods*: a total of 301 patients diagnosed with STEMI treated with primary percutaneous coronary intervention (pPCI) were included in the study. Reinfarction, revascularization, cerebrovascular event, and cardiac death were determined as major cardiac clinical events. The follow-up period of our study was 475 ± 193 days. *Results*: Univariate analysis revealed that factors influencing delay time included BMI, hypertension diabetes, smoking habit and variability in pain intensity. In multivariate logistic regression analysis, BMI, diabetes, hypertension, smoking, variation in pain intensity, and infarct-related artery other than the LAD were identified as independent factors associated with increased delay times. We determined the cut-off values predicting the composite endpoint as 122.5 min for patient delay, 95.5 min for system delay, and 371 min for total ischemic time. It was observed that the in-hospital NT pro-BNP values of the patients presenting early were lower (181 vs. 594 pg/mL *p* < 0.001), had a higher ejection fraction at the first measurement, and even improved at the sixth week of follow-up (*p* = 0.047). *Conclusions*: Prolonged ischemia duration was associated with several factors. Early reperfusion in STEMI patients reduces both cardiac death and clinical events. Delays are influenced by patient awareness, emergency care efficiency, and hospital-specific factors. Improving education, response times, and hospital protocols is essential to minimize delays and improve outcomes.

## 1. Introduction

Immediate recanalization of the infarct related artery (IRA) has been the major goal of treatment for patients with ST-segment elevation myocardial infarction (STEMI) due to consistently demonstrated improved clinical outcomes with early reperfusion. Blood flow restoration ensured via primary percutaneous coronary intervention (pPCI) within 120 min of diagnosis is superior to fibrinolytic therapy in reducing death, reinfarction, revascularization, and stroke [1,2]. Every 30 min of delay in reperfusion, increases the relative risk of one-year mortality by 7.5% [3]. Hence, despite the target of 120 min, tissue reperfusion should be achieved as soon as possible. Shortening of the ischemic duration was strongly correlated with reduced mortality, independent of confounding variables [4]. The total ischemic time consists of symptom onset to medical contact time, which depends on patient-oriented factors, and diagnosis to recanalization of IRA duration, which is mostly related to the emergency medical care system. Although the latter is well defined and current guidelines recommend strategies to shorten this period, the literature consists of conflicting data regarding the delay caused by patient-related factors. By implementing emergency care system policies to shorten ischemic duration, a significant decrease in system-related delay lengths has been observed; however, patient-related delay lengths remained unchanged over the last decades in the USA and Europe [5,6]. Female sex, age, non-white race, low household income, geographic regions, and medical history of previous angina, diabetes, or hypertension, were associated with a prehospital delay in previous studies [7,8,9].

This study was designed to determine the factors that cause patient delay, system delay, and total ischemic time in patients admitted to the hospital with STEMI. In addition, the effect of this delay on the patient’s prognosis has also been investigated.

## 2. Methods

The study was conducted in a university hospital cardiology clinic that has a 24/7 serving high-volume pPCI facility (>400 STEMI patients annually). The study protocol was approved by the local ethics committee of our institution (date: 17 October 2018/number: 30330229-604.01.02-76606). Patients were prospectively enrolled in the study after informed consent was obtained between November 2018 and February 2020. Written informed consent was obtained from each patient or their relatives following a detailed explanation of the objectives and protocol of the study.

### 2.1. Study Population

Patients diagnosed with STEMI according to current guidelines and treated with pPCI were included in the study. The exclusion criteria were as follows: (i) under 18 years old; (ii) refused to give informant consent; (iii) treated with emergency coronary artery bypass grafting surgery; (iv) unable to attend scheduled visits; (v) life expectancy of <1 years due to concomitant noncardiac comorbidities; (vi) patients whose ischemic duration cannot be determined. Most of the patients in this study population were admitted firstly to another state hospital and had a diagnosis of STEMI, then transferred to our institution by ambulances from the national central emergency healthcare system of the Ministry of Health. A small portion of the patients were those who applied directly to our hospital. Thus, our final study population consisted of 301 STEMI patients. Demographic data of each patient, concomitant diseases, family history of premature cardiovascular disease, and medications on the day of STEMI were recorded. Ages were categorized as <40, 40–65, and >65 years and BMI was categorized as cut-off values for normal weight, increased weight, and obesity for descriptive purposes. This study was conducted in the country’s largest economic hub, specifically in Istanbul. The per capita income in Istanbul ranges between 15,000 and 20,000 USD, whereas the national average is between 10,000 and 11,000 USD.

Patient delay was defined as the time between symptom onset and first medical contact, which was either an emergency service call or an emergency department admission. First medical contact time is accepted as written on the barcode at the first hospital the patient attended, thus more objective data are obtained. System delay was defined as the time between first medical contact and restoration of distal coronary flow via pPCI. The total ischemic duration was calculated as prehospital delay plus system delay. Blood samples were collected immediately after the pPCI during the coronary care unit (CCU) stay.

The cut-off values for patient delay, system delay, and total ischemic time were determined using ROC curve analysis based on their association with the composite endpoint of major adverse cardiac events (reperfusion, re-infarction, cerebrovascular events, and cardiac death). These thresholds (122.5 min, 95.5 min, and 371 min, respectively) were chosen to facilitate the identification of patients at increased risk and were subsequently used as binary variables in logistic regression models to explore factors contributing to treatment delays.

The term “Variation in Pain Intensity” refers to the persistence of the patient’s pain or the fluctuating intensity of the pain, where it alternates between periods of worsening and relief.

### 2.2. Coronary Angiography

All coronary angiographies were performed using a Philips Allura Xper angiography machine (Philips, Andover, MA, USA). Patients were referred directly to the catheter laboratory and, following diagnostic angiography, revascularization of the culprit artery was completed with a stent implantation. However, in patients with multivessel disease who were deemed suitable for a CABG to ensure complete revascularization, a pPCI procedure was completed after balloon dilatation in the case that TIMI III distal flow ensured. All the pPCI procedures were performed by two experienced invasive cardiologists and images were reviewed by another senior cardiologist. Only culprit artery revascularization was performed at the index procedure and revascularization of significant lesions (>90% luminal narrowing) at the nonculprit arteries before hospital discharge.

### 2.3. Echocardiographic Evaluation

All patients underwent echocardiographic investigation at least twice during index hospitalization; prior to pPCI to exclude mechanical complications as well as an assessment of wall motion abnormalities and comprehensive echocardiographic evaluation before hospital discharge using Philips IE-33 and Philips EPIQ echocardiography machines (Philips, Andover, MA, USA) by two experienced cardiologists and a control echocardiographic evaluation during the sixth week of post-STEMI. The assessment of wall motion abnormalities and valvular functions and the measurement of the diameter and volumes of the heart chambers were performed as recommended by current guidelines. Left ventricular ejection fraction was calculated using biplane (apical 4- and 2-chamber view) modified Simpsons’ method.

### 2.4. Follow-Up

All patients were followed-up in a dedicated outpatient clinic. The visit schedule—during the sixth week post-infarction, the sixth month, the first year, and annually thereafter—was explained to each patient prior to hospital discharge. Adherence to medical therapy, dose titration, and the functional status of each patient were assessed. New-onset heart failure was diagnosed when symptoms of heart failure were accompanied by elevated natriuretic peptide or signs of volume overload. Cardiac death was decided according to National Death Declaration System records obtained from the Ministry of Health. Reinfarction, revascularization, cerebrovascular event, and cardiac death were determined as major cardiac clinical events. The follow-up period of our study was 475 ± 193 days (longest follow-up period was 838 days).

### 2.5. Statistical Analysis

Statistical analysis was performed using SPSS version 21 (SPSS Inc., Chicago, IL, USA). In descriptive analyses, the number and percentage in categorical variables, mean ± standard deviation, or median 25–75th percentiles values were given where appropriate. The normality of the continuous variables was demonstrated via the Kolmogorov–Smirnov test, the coefficient of variation, histogram, and Q–Q plot. Comparisons between the groups were evaluated with the Mann–Whitney U test for continuous variables and with Chi-squared test for categorical variables. The correlation between continuous data was examined by the Spearman correlation test. In determining the cut-off values of the delay time of the patients included in the study, it was evaluated by ROC analysis according to whether it was the composite endpoint (at least one of reperfusion, reinfarction, cerebrovascular event, and cardiac mortality). To identify the factors that increase the risk of delay of patient, system, and total ischemic time, variables with a *p*-value below 0.250 in univariate analysis were assessed with binary logistic regression analysis. The backward LR method was used in multivariable regression analysis. A *p*-value below 0.05 was considered significant.

## 3. Results

From 2018 to 2020, 301 patients with STEMI (mean age 55.7 ± 10.2, 85.7% male) who fulfilled the inclusion–exclusion criteria were included. The baseline characteristics of the patients are summarized in Table 1. Hypertension (25.9%) was the most common concomitant disease according to anamnesis and/or drugs used. Among the risk factors of atherosclerosis, the most common was smoking (69.4%).

In the univariate analysis of the factors that determine the system delay time according to the recommended target time of 120 min in the “2023 ESC Guidelines for the management of acute coronary syndromes”, the education level, diabetes, and variability in pain intensity were found to affect this duration. In multivariate logistic regression analysis, diabetes, variation in pain intensity, and infarct-related artery other than LAD were found as the independent factors that increased delay times.

**Figure 1:**
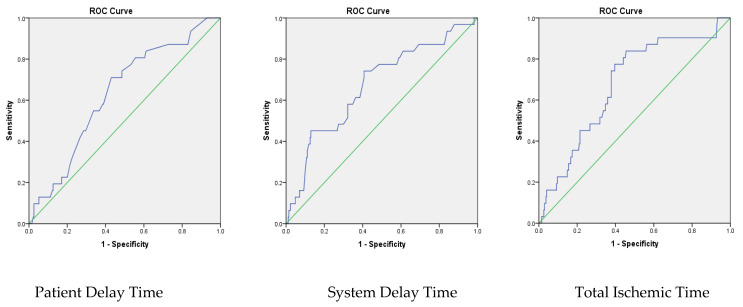
ROC curves.

**Chart 1:**
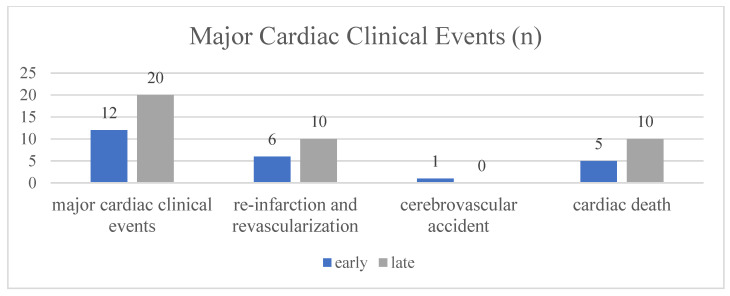
Major Cardiac Clinical Events in Early and Late Group According to Total Ischemic Time.

The combined endpoint of major cardiac clinical events occurred in 32 (10.6%) and cardiac death occurred in 15 (4.9%) patients. ROC analysis was performed with the delay times of the patients who were included in the study (Table 2, Figure 1). We considered reperfusion, reinfarction, and a cerebrovascular event as the composite endpoint as well as cardiac mortality. We determined the cut-off values predicting the composite endpoint as 122.5 min for patient delay, 95.5 min for system delay, and 371 min for total ischemic time. According to the cut-off value of the ischemic times obtained by ROC analysis, the patients were divided into two groups as early and late presenters. The numbers of major cardiac clinical events in the early and late group according to the total ischemic time are given in Chart 1. Major cardiac clinical events occurred in 12 patients in the early group and 20 patients in the late group (*p* = 0.008). During follow-up, reinfarction and revascularization were seen in 6 patients in the early group and 10 patients in the late group. There was only one cerebrovascular accident in a patient who presented early. Hence, nonfatal cardiovascular events were similar between groups (*p* = 0.066 and 0.409). However, cardiac death was significantly lower in the early group (5 patients in the early group and 10 patients in the late group) (*p* = 0.034).

The univariate and multivariate analysis of the factors determining early and late presenters is summarized in Table 3. In univariate analysis, while the patient delay time was significantly longer in patients with diabetes mellitus, intermittent angina pectoris, and EF < 40%, it was shorter in patients with hypertension. BMI, diabetes mellitus, smoking, intermittent angina pectoris, culprit vessel other than left anterior descending artery, and multivessel disease were significantly associated with system delay time. A high level of education, diabetes mellitus, intermittent angina pectoris, and multivessel disease were significantly associated with total ischemic time. In multivariate analysis, the factors affecting the patient delay time were hypertension, intermittent angina pectoris, EF < 40%, and culprit vessel other than LAD. BMI, previous admission to hospital with angina pectoris, intermittent angina pectoris, EF < 40%, and culprit vessel other than LAD were significantly associated with system delay time. The level of education, diabetes mellitus, intermittent angina pectoris, and culprit vessel other than LAD were significantly associated with total ischemic time.

It was observed that the in-hospital NT pro-BNP values of the patients presenting early were lower (median 181 vs. 594 pg/mL *p* < 0.001), had a higher ejection fraction at the first measurement, and even improved in the sixth week of follow-up (*p* = 0.047). According to the correlation analysis between NT pro-BNP level and delay times, there was a weak positive correlation between patient delay (r: 0.287 *p*: 0.002), system delay (r: 0.374 *p*: <0.001), and total ischemic time (r: 0.324 *p* = 0.001) and NT pro-BNP. A weak negative correlation was found between the ejection fraction measured in the echocardiographic examination performed after the procedure and patient delay time (r: −0.274, *p* = 0.01) and total ischemic time (r: −0.280, *p* = 0.02).

## 4. Discussion

In this study, we present the data from the prospective single-center study that aims to reveal the factors affecting ischemic times and to determine how much the left ventricle is affected and MACCE according to these times. We found that ischemic times are a prognostic marker and MACCE incidence is associated with ischemic time. Our results show that the factors affecting the delay times are hypertension, diabetes mellitus, intermittent angina pectoris, EF < 40%, culprit vessel other than LAD, multivessel disease, BMI, smoking, previous admission to hospital with angina, and level of education.

The cause of prolonged ischemic time is a general problem independent of the person and nation. In addition, it has also been reported that the total ischemic time is greater than 4 h in up to half of the patients, which is an independent predictor of MACCE [10]. In our study, diabetes was determined as a factor that increased all ischemic times in univariate analysis. Hypertension was a factor that shortened the patient delay time. A smoking habit was found to shorten system duration in univariate analysis, probably due to increased awareness of atherosclerosis in terms of physicians. In another study conducted during the COVID-19 pandemic in Turkey, the factors associated with prolonged patient delay were hypertension, diabetes, gender, age, and a smoking habit [11]. Trials from developed countries such as Denmark and Austria have also confirmed that the prolonged patient delay durations are associated with gender and diabetes [12,13].

Presenting with multivessel disease and variability of pain severity were identified as prolonging factors. The reason for the prolongation of the durations in patients with multivessel disease can be explained by ischemic preconditioning. In recurrent ischemic attacks, all of the following have the possibility of being life threatening: arrhythmia, the severity of angina, and the magnitude of ST segment elevation decrease [14,15]. The fact that the ejection fraction measured at the first admission is below 40% is one of the factors that increases the patient delay time and system delay time. We thought that an earlier arrival of patients with anterior myocardial infarction caused by an occlusion in the LAD artery was related to more common ECG findings and more extensive ischemic findings. The variability of pain intensity causing admission delays may be related to the fact that the patients underestimate the symptoms and do not disturb the emergency department unless they are “really ill” [16,17,18].

According to the 2019–2020 education data of the national statistical institution, the university and above education level was 43.4%, while it was found to be 8.3% in our study. It was observed that a higher education level decreased the system delay time—suggested by guideline—and total ischemic time [19].

We also found that with a prolongation of delay times, indicators of extensive myocardial injury, such as decreased LVEF and higher NT pro-BNP levels are more common. Ramakrishnan et al. observed that high NT pro-BNP values were associated with a longer total ischemic time and multivessel disease [20]. Moreover, we present that late presenters had lower EF on the day of the event and improved less during follow-up. An important explanation for our findings is that the infarct size is significantly affected by the duration of coronary occlusion. Therefore, late reperfusion is expected to result in less myocardial salvage and a higher mortality rate than found with early reperfusion.

The aim of an invasive strategy in STEMI is to restore myocardial flow as soon as possible from symptom onset. The prognostic role of time to therapy in patients with STEMI treated with pPCI was demonstrated [21,22]. The contemporary data indicate that in-hospital mortality rates in patients with STEMI are 4–12% and the mortality rate in the first year is around 7–10% [23,24,25]. In our study, cardiac death was observed in 15 (4.9%) patients and major cardiac clinical events were observed in 32 (10.6%) patients during 475 ± 193 follow-up days in 301 patients. Reperfusion of the infarct related artery in the early period decreased the risk of cardiac death [26,27,28]. De Luca et al. demonstrated that every minute of delay in primary angioplasty for STEMI affects 1-year mortality, even after adjustment according to the baseline characteristics [3]. Rathore et al., who investigated the relationship between door-balloon time and mortality, confirmed that the relationship between system delay and mortality increased linearly [29]. The trials from Poland and Egypt also confirmed the linear relationship between total ischemic time and long-term mortality [30,31]. In an FITT-STEMI study, the effect of delay times was investigated in hemodynamically unstable patients and each 10-min delay caused 3.31 additional deaths, especially in patients with cardiogenic shock [32].

Another study from China confirms that health system improvements can shorten ischemic time and have better outcomes on prognosis [33]. In this study, we emphasized the effect of time on clinical outcomes and clinical features that prolong the time. In our opinion, every country should improve its own health system with studies like this and increase information programs in order to shorten the ischemic duration of ST-elevation myocardial infarction. The structured solutions tailored to their individual, local characteristics, and resources are a vital need.

## 5. Limitations

Our study has been conducted as a single center one, which may cause an unrecognized bias normally avoided in multicenter analyses. However, this secured uniformity of algorithms of care and could have increased completeness of data.

Another limitation is that the patient population is restricted to STEMI patients as defined by the guidelines, with no inclusion of currently recognized OMI or very high-risk NSTEMI cases.

Due to the COVID-19 pandemic, which affected the whole world, the inclusion of patients was discontinued in March 2020, the start date of the pandemic in Turkey. On the other hand, some patients were also excluded from the study because their visits were delayed due to the pandemic situation.

## 6. Conclusions

In conclusion, we determined the cut-off values predicting the composite endpoint as 122.5 min for patient delay, 95.5 min for system delay, and 371 min for total ischemic time. The affected ischemic time was associated with hypertension, diabetes mellitus, intermittent angina pectoris, EF < 40%, culprit vessel other than LAD, multivessel disease, BMI, smoking, previous admission to hospital with angina, and level of education.

In STEMI patients, reperfusion should be achieved within 120 min as specified in the guidelines. However, most patients cannot apply to the hospital in this golden time window. With this study, we wanted to investigate the causes of the delay time in our region and to determine their relationship with cardiac death. Also, we presented that not only cardiac death but also the cardiac clinical events were decreased with reperfusion of the infarct related artery in the early period.

Patient education regarding the alarm signs of myocardial infarction is crucial in reducing patient-dependent delay times in seeking medical care. By increasing awareness of symptoms such as chest pain, shortness of breath, and dizziness, patients can be encouraged to seek timely medical attention, which can significantly shorten delays in treatment. Such an intervention could directly improve clinical outcomes, as prompt intervention in STEMI cases is key to minimizing myocardial damage and improving survival rates.

This study suggests that, although many thresholds for ischemic times have been reported in the literature, probably the best approach would be for each center performing 24/7 primary percutaneous coronary intervention to examine its own times and make cause-oriented plans to shorten these times, for health-related government agencies to review public information programs to shorten patient delay times, and for emergency health care units to make arrangements to shorten system delay times.

## Figures and Tables

**Table 1:** Patient’s Baseline and Clinical Characteristics and Univariable and Multivariable Logistic Regression Analyses of Variables That Affect Delay Time (*Suggested Duration in 2023 STEMI Guidelines*).

Patient Characteristics	All	System Delay Time > 120 min	
		Univariate	Multivariate
Age	55.7 ± 10.2	1.01 (0.99–1.03)	
Male (%) *(Reference: Female)*	258 (85.7)	1.32 (0.85–2.03)	
BMI (kg/m^2^)	27.1 (26.8–27.6)	0.99 (0.93–1.06)	**1.10 (1.02–1.86)**
High School Education (%) *(Reference: Primary and below)*	77 (25.5)	**0.86 (0.74–0.99)**	
University And Above Education (%) *(Reference: Primary and below)*	25 (8.3)	**0.82 (0.68–0.98)**	
Married (*Reference: Single*) (%)	252 (83.7)	0.98 (0.81–1.21)	
Hypertension (%)	78 (25.9)	0.89 (0.61–1.31)	
Diabetes Mellitus (%)	62 (20.5)	**2.54 (1.64–3.91)**	**2.65 (1.45–4.84)**
Dyslipidaemia (%)	30 (9.9)	0.78 (0.47–1.31)	
Family History of MI (%)	135 (44.8)	1.14 (0.81–1.63)	
History of Previous MI (%)	19 (6.3)	0.93 (0.47–1.86)	
Congestive Heart Failure (%)	8 (2.6)	2.41 (0.38–15.16)	
Alcohol (%)	100 (33.2)	1.34 (0.91–2.01)	
Smoking (%)	209 (69.4)	1.21 (0.84–1.74)	
Coronary Artery Disease (%)	48 (15.9)	0.87 (0.56–1.36)	
Chronic Renal Failure (%)	12 (3.9)	0.69 (0.34–1.39)	
Chronic Obstructive Pulmonary Disease (%)	14 (4.6)	2.12 (0.58–7.74)	0.13 (0.02–1.13)
**Situation From Onset to Admission**			
Ambulance (112) Transportation to The Hospital	93 (30.8)	0.91 (0.78–1.06)	
Previous Admission to Hospital with Angina	106 (35.2)	1.12 (0.77–1.64)	
Variation in Pain Intensity *	176 (58.4)	**1.28 (1.11–1.48)**	**2.48 (1.39–4.39)**
**Angiographic Lesion Characteristics**			
Culprit Vessel Other Than LAD	165 (54.8)	1.41 (0.98–2.04)	1.60 (0.93–2.76)
Multivessel Disease (*Reference: single vessel*)	131 (43.5)	1.13 (0.97–1.32)	
**Outcome Variables**			
Ejection Fraction < 40%	108 (35.8)		
Total Cholesterol (mg/dL)	177 (176.6–185.9)		
Triglyceride (mg/dL)	137.5 (148.8–173.8)		
LDL-Cholesterol (mg/dL)	125 (122.4–130.6)		
HDL-Cholesterol (mg/dL)	38 (37.8–39.9)		
Hba1c (%)	5.9 (6.3–7.1)		
Creatinine (mg/dL)	0.82 (0.85–0.93)		
Final TIMI Flow 3	276 (91.6)		

* The term “Variation in Pain Intensity” is referred to the persistence of the patient’s pain or the fluctuating intensity of the pain, where it alternates between periods of worsening and relief. Abbreviations: BMI: body mass index, MI: myocardial infarction, LDL: low density lipoprotein, HDL: high density lipoprotein, LAD: left anterior descending artery, TIMI: thrombolysis in myocardial infarction.

**Table 2:** ROC analysis of the ischemic time.

	Area Under the Curve (95% Confidence Interval)	Cut-Off Value (min)	*p*-Value
Patient delay time	0.632 (0.536–0.762)	122.5	0.016
System delay time	0.672 (0.569–0.775)	95.5	0.002
Total ischemic time	0.677 (0.581–0.772)	371	0.001

**Table 3:** **Univariable And Multivariable Logistic Regression to Variables That Affect Delay Time**.

Patient Characteristics	Patient Delay Time > 122.5 min		System Delay Time > 95.5 min		Total Ischemic Time > 371 min	
	Univariate	Multivariate	Univariate	Multivariate	Univariate	Multivariate
Age (years)	1.01 (0.99–1.03)		1.02 (0.99–1.04)		0.99 (0.97–1.02)	
Man	1.05 (0.54–2.03)		0.95 (0.49–1.84)		0.93 (0.41–2.12)	
BMI (kg/m^2^)	0.99 (0.93–1.06)		**1.08 (1.01–1.15)**	**1.08 (1.01–1.16)**	1.01 (0.93–1.09)	
High School Education (%)	0.68 (0.39–1.78)		0.63 (0.36–1.09)		0.43 (0.76–1.51)	
University And Above Education (%)	0.49 (0.19–1.23)		0.58 (0.24–1.41)		0.55 (0.23–2.19)	
Married *(Reference: Single)*	1.49 (0.78–2.86)		1.11 (0.59–2.08)		0.94 (0.44–2.02)	
Hypertension (%)	**0.48 (0.27–0.85)**	**0.37 (0.19–0.71)**	1.08 (0.64–1.83)		0.54 (0.26–1.10)	
Diabetes Mellitus (%)	**1.92 (1.09–3.37)**	**2.12 (1.04–4.34)**	**2.22 (1.26–3.92)**	1.74 (0.97–3.13)	1.64 (0.86–3.12)	
Dyslipidaemia (%)	1.14 (0.53–2.44)		1.12 (0.52–2.39)		1.28 (0.52–3.15)	
Family History of MI (%)	1.07 (0.68–1.71)		0.94 (0.59–1.49)		1.05 (0.59–1.85)	
History of Previous MI (%)	0.85 (0.32–2.22)		0.84 (0.32–2.18)		0.76 (0.21–2.69)	
Congestive Heart Failure (%)	0.88 (0.21–3.74)		0.20 (0.02–1.65)		0.58 (0.07–4.79)	
Alcohol (%)	1.09 (0.67–1.78)		0.79 (0.48–1.29)		1.04 (0.57–1.89)	
Smoking (%)	1.09 (0.66–1.79)		**0.55 (0.33–0.9)**	**0.59 (0.34–0.99)**	1.53 (0.79–2.95)	1.93 (0.93–4.00)
Coronary Artery Disease (%)	0.95 (0.51–1.79)		1.04 (0.56–1.95)		0.79 (0.35–1.78)	
Chronic Renal Failure (%)	1.05 (0.33–3.39)		1.04 (0.32–3.34)		0.22 (0.42–2.59)	
Chronic Obstructive Pulmonary Disease (%)	0.23 (0.05–1.06)	0.27 (0.05–1.39)	0.79 (0.26–2.43)		41803 (0–NA)	
**Situation From Onset to Admission**						
Ambulance (112) Transportation to The Hospital *(Reference: Herself/Himself)*	0.64 (0.39–1.07)		0.77 (0.47–1.28)		0.57 (0.29–1.12)	
Previous Admission to Hospital with Angina	1.13 (0.70–1.83)		0.72 (0.44–1.18)	0.63 (0.37–1.06)	0.85 (0.46–1.55)	
Variation In Pain Intensity	**9.39 (5.19–17.01)**	**9.95 (5.32–18.58)**	**2.14 (1.32–3.46)**	**2.34 (1.39–3.93)**	**60.95 (8.31–447)**	**62.29 (8.45–459.34)**
**Angiographic Lesion Characteristics**						
Culprit Vessel Other Than LAD *(Reference LAD)*	1.41 (0.88–2.25)	**1.76 (1.01–3.08)**	**1.81 (1.13–2.90)**	**1.81 (1.09–2.99)**	1.37 (0.77–2.45)	
Multivessel Disease *(Reference: Single Vessel)*	**1.85 (1.16–2.94)**	1.65 (0.95–2.87)	**1.61 (1.01–2.56)**		**2.42 (1.35–4.34)**	**2.61 (1.36–5.00)**
		R^2^: 0.145		R^2^: 0.179		R^2^: 0.346

Abbreviations: BMI: body mass index; MI: myocardial infarction; LDL: low-density lipoprotein; HDL: high-density lipoprotein; LAD: left anterior descending artery; TIMI: thrombolysis in myocardial infarction.

## Data Availability

The data are available upon reasonable request from the authors.
